# Characterization of the biosynthetic gene cluster for cryptic phthoxazolin A in *Streptomyces avermitilis*

**DOI:** 10.1371/journal.pone.0190973

**Published:** 2018-01-11

**Authors:** Dian Anggraini Suroto, Shigeru Kitani, Masayoshi Arai, Haruo Ikeda, Takuya Nihira

**Affiliations:** 1 International Center for Biotechnology, Osaka University, Suita, Osaka, Japan; 2 Graduate School of Pharmaceutical Sciences, Osaka University, Suita, Osaka, Japan; 3 Kitasato Institute for Life Sciences, Kitasato University, Sagamihara, Kanagawa, Japan; 4 MU-OU Collaborative Research Center for Bioscience and Biotechnology, Faculty of Science, Mahidol University, Bangkok, Thailand; Universite Paris-Sud, FRANCE

## Abstract

Phthoxazolin A, an oxazole-containing polyketide, has a broad spectrum of anti-oomycete activity and herbicidal activity. We recently identified phthoxazolin A as a cryptic metabolite of *Streptomyces avermitilis* that produces the important anthelmintic agent avermectin. Even though genome data of *S*. *avermitilis* is publicly available, no plausible biosynthetic gene cluster for phthoxazolin A is apparent in the sequence data. Here, we identified and characterized the phthoxazolin A (*ptx*) biosynthetic gene cluster through genome sequencing, comparative genomic analysis, and gene disruption. Sequence analysis uncovered that the putative *ptx* biosynthetic genes are laid on an extra genomic region that is not found in the public database, and 8 open reading frames in the extra genomic region could be assigned roles in the biosynthesis of the oxazole ring, triene polyketide and carbamoyl moieties. Disruption of the *ptxA* gene encoding a discrete acyltransferase resulted in a complete loss of phthoxazolin A production, confirming that the *trans*-AT type I PKS system is responsible for the phthoxazolin A biosynthesis. Based on the predicted functional domains in the *ptx* assembly line, we propose the biosynthetic pathway of phthoxazolin A.

## Introduction

Polyketides and non-ribosomal peptides are structurally diverse classes of natural products representing important therapeutic and agricultural chemicals [1,2]. In bacteria, polyketides are typically produced by polyketide synthase (PKS) assembly lines that are composed of functional modules. Each module is responsible for one step of chain extension of the growing products and consists of a set of domains which dictate the chemical functionality of the incorporated building block [3,4]. The minimal domains of a PKS module are an acyltransferase (AT), ketosynthase (KS) and acyl carrier protein (ACP) domain, where the AT domain loads a coenzyme A-activated dicarboxylic acid extender unit onto the ACP domain and the KS domain of the preceding domain catalyzes condensation of the nascent polyketide with the extender unit. Meanwhile, nonribosomal peptide synthetase (NRPS) also forms multienzyme assembly lines that are similar to those of PKS, and produces peptide-containing compounds [5]. In the NRPS module, an adenylation (A) domain activates an amino acid, and loads onto a peptidyl carrier protein (PCP). Subsequently, a condensation (C) domain generates an amide bond with the growing chain tethered to the upstream PCP domain. Many of the bacteria sequenced to date have been found to possess a “hybrid” PKS/NRPS enzyme [6] and show that both PKS and NRPS function simultaneously in the same assembly line [7], but the products synthesized by most of the hybrid PKS/NRPS enzymes are still unknown.

Oxazole-containing polyketides are typical PKS/NRPS hybrid products, and have various biological activities for medicinal and agricultural purposes; e.g., oxazolomycin is an oxazole-triene antibiotic of *Streptomyces albu*s JA3453 showing antibacterial and antitumor activities [8, 9], rhizoxin is a methyl-oxazole macrolide from the bacterial endosymbiont *Burkholderia rhizoxina* [10], and conglobatin is an unusual symmetrical macrolide from *Streptomyces conglobatus* showing antitumor activity [11]. Within these oxazole-containing polyketides, oxazolomycin and rhizoxin are synthesized by the *trans*-AT type I PKS system together with NRPS machinery [10,12]. One distinct feature of the *trans*-AT type I PKS system is that the PKS modules lack an AT domain and one or a few free-standing ATs provide extender units for each elongation step [13,14]. This type of PKS modular system has contributed to the expansion of complexity in the polyketide biosynthetic machinery.

**Fig 1:**
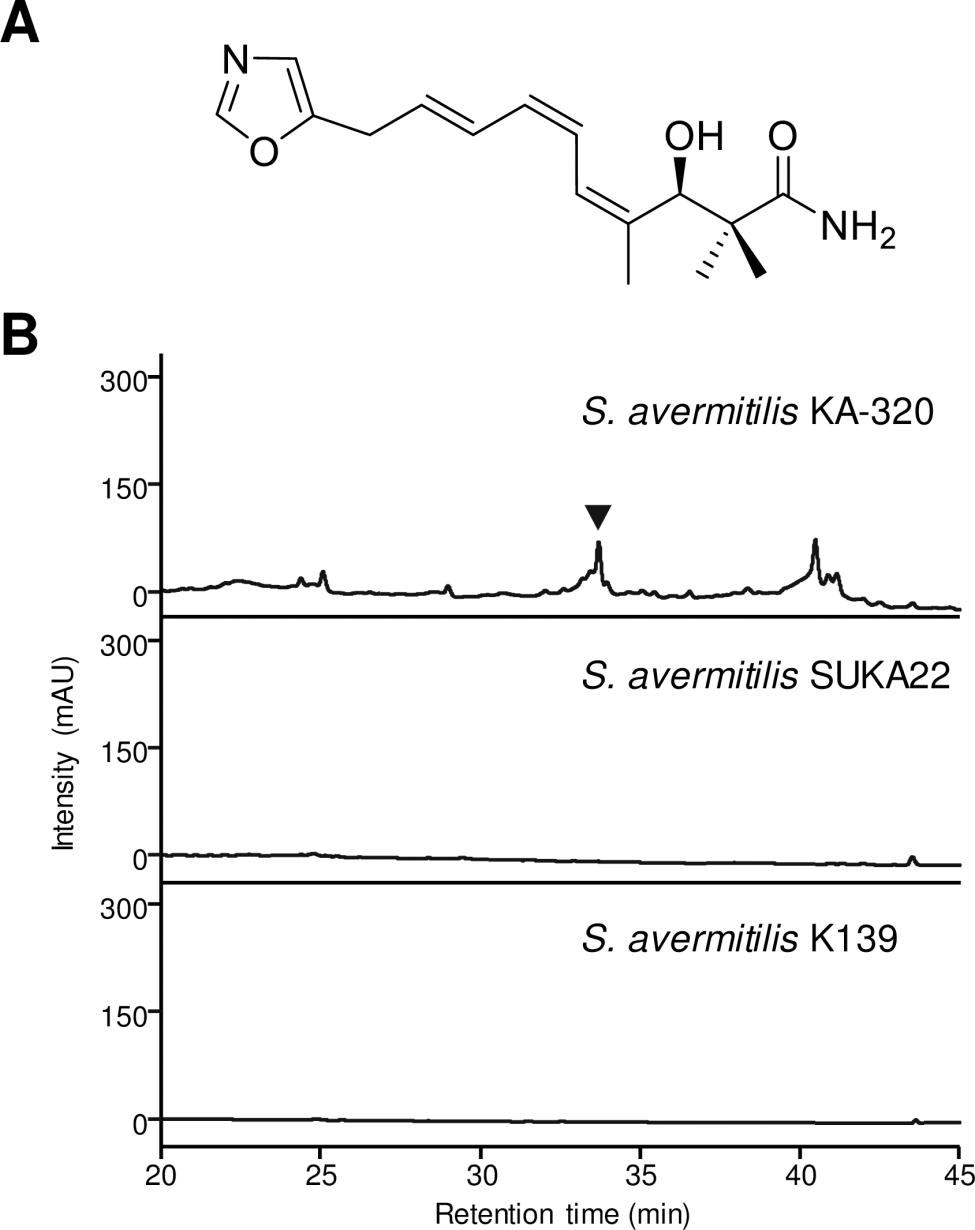
Phthoxazolin A production in the *S*. *avermitilis* progeny. (A) Chemical structure of phthoxazolin A. (B) HPLC chromatograms of MeOH extracts from *S*. *avermitilis* KA-320 (top), *S*. *avermitilis* SUKA22 (middle), and *S*. *avermitilis* K139 (bottom). mAU, milliabsorbance units at 275 nm. Phthoxazolin A was detected at a retention time of 33.9 min, and is indicated by an inverted triangle.

Phthoxazolin A (Fig 1A), one of the oxazole-containing polyketides, is an inhibitor of cellulose biosynthesis and exerts growth-inhibitory activity against plant pathogenic oomycetes [15–17]. The structure of phthoxazolin A includes a unique 5-substituted oxazole ring connected to a triene moiety, which corresponds to the substructure of oxazolomycin. We have previously demonstrated that phthoxazolin A is a cryptic metabolite of *Streptomyces avermitilis*, the producer of the important anthelmintic agent avermectin [18]. Although the *in silico* analysis predicted that the genome of *S*. *avermitilis* harbors at least 38 gene clusters for secondary metabolites [19], there is no assembly line similar to that for oxazolomycin biosynthesis. Thus, the biosynthetic gene cluster for phthoxazolin A would be hidden somewhere in the *S*. *avermitilis* genome.

In this study, we performed genome sequencing, comparative genomic analysis, and mutagenesis to identify a biosynthetic gene cluster for phthoxazolin A, and demonstrated that the gene cluster is laid on the extra genomic region of the original avermectin producer, and phthoxazolin A is synthesized by the *trans*-AT type I PKS system. The proposed assembly line for phthoxazolin A biosynthesis also suggests a new cleavage system in the PKS/NRPS machinery.

## Materials and methods

### Bacterial strains, plasmids, and growth conditions

*S*. *avermitilis* KA-320 (isogenic to MA-4680, ATCC 31267 and NRRL 8165) [20], K139 (a progeny of KA-320) [21], SUKA22 (K139 as a genetic background) [22] strains were obtained from the culture collection of Kitasato Institute, and *S*. *avermitilis* KA-320 Δ*avaR3* [20] was used in this study. All these strains were grown on YMS-MC medium for spore formation [20]. *Escherichia coli* DH5α was used for general DNA manipulation, and *E*. *coli* F^-^*dcm* Δ(*srl-recA*) *306*::Tn*10* carrying pUB307-*aph*::Tn*7* was used for *E*. *coli/Streptomyces* conjugation. The plasmids pKU451, pKU470, pKU479, pKU480, and pKU250 were used to construct a vector for gene deletion [23]. The media and general *E*. *coli* and *Streptomyces* manipulations were as described previously [20]. For analysis of phthoxazolin A production, spores (1.0 X 10^8^ CFU) of the *S*. *avermitilis* strains were inoculated into 70 mL APM medium in a 500-mL baffled flask, and mycelia were harvested after 48 h of cultivation. The mycelia were washed, re-suspended in fresh APM medium and stored at -80°C until use as a seed culture. All the primers are listed in S1 Table.

### Analysis of phthoxazolin A production

The seed culture was inoculated on 2.5 mL YMD solid medium [18], followed by incubation at 28°C for 3 days. The agar culture was diced and extracted with an equal volume of methanol. The methanol extract was analyzed by using a HPLC system as described previously. [18].

### Construction of *S**treptomyces* *a**vermitilis* large-deletion (SALD) mutant strains

For the SALD-1 mutant, two regions (position 77,219–79,457 nt and 79,479–81,744 nt) were PCR-amplified by the primer pairs (sav68-up*-*Fw/sav68-up-Re and sav71-dw*-*Fw/sav71-dw*-*Re). These fragments were digested by *Hin*dIII and *Spe*I, and inserted to pKU451 resulting in pLT143. This plasmid was digested with *Spe*I and ligated together with a fragment containing a hygromycin B phosphotransferase gene (*hph*) with mutant *loxP* sequence (mut-*loxP*) (PCR-amplified with pKU480 and the mutloxP-SpeI-Fw/wo-mutloxP-SpeI-Re primer pair) to generate pLT144. A 6.6 kb *Hin*dIII fragment, recovered from pLT144, was cloned into pKU250 at the *Hind*III site to obtain pLT145. pLT145 was introduced by intergeneric conjugation to *S*. *avermitilis* KA-320 Δ*avaR3* mutant to yield *S*. *avermitilis* Δ*avaR3*/*sav71*::mut-*loxP*-*hph* mutant. Another two regions (582,859–584,876 nt and 587,127–584,906 nt) were also PCR-amplified by the primer pairs (sav432-up-Fw/sav434-up-Re and sav434-dw*-*Fw/sav434-dw-Re). These fragments were treated with *Hin*dIII/*Spe*I, and ligated to pKU451 to get pLT146. A kanamycin-resistant gene (*aphII*) with mut-*loxP* at downstream end was PCR-amplified by the primer pair wo-mutloxP-SpeI-Fw/ mutloxP-SpeI-Re using pKU479 as a template, and introduced into the *Spe*I site of pLT146 to generate pLT147. A 6.0 kb *Hin*dIII fragment, recovered from the resultant plasmid, cloned into at the *Hin*dIII site of pKU250 to obtain pLT148. pLT148 was introduced to *S*. *avermitilis* Δ*avaR3*/*sav71*::mut-*loxP*-*hph* mutant, resulting in *S*. *avermitilis* Δ*avaR3*/*sav71*::mut-*loxP*-*hph* mutant/Δ*sav434*::*aphII*-mut-*loxP*. The *cre* expression plasmid pKU470 was introduced into the strain for removal of a 0.51 Mb region covering from *sav71* to *sav434*.

For the SALD-2 mutant, the *aphII* gene of pLT148 was replaced with a *hph* gene harboring mut-*loxP* by *Spe*I digestion and ligation, resulting in pLT149. pLT149 was introduced into *S*. *avermitilis* KA-320 Δ*avaR3* mutant to yield *S*. *avermitilis* Δ*avaR3*/*sav434*::mut-*loxP*-*hph* mutant. Two regions (999,468–997,573 nt and 1,004,009–1,002,110 nt) were PCR-amplified by the primer pairs (sav845-up*-*Fw/sav845-up-Re and sav845-dw*-*Fw/sav845-dw*-*Re), and ligated with pKU451 to get pLT150. This plasmid was ligated with the *aphII* gene prepared previously to generate pLT151. A 5.4 kb *Hin*dIII fragment, recovered from pLT151, was cloned into pKU250 at the *Hin*dIII site to obtain pLT152. pLT152 was introduced to *S*. *avermitilis* Δ*avaR3*/*sav434*::mut-*loxP*-*hph* mutant, resulting in *S*. *avermitilis* Δ*avaR3*/*sav434*::mut-*loxP*-*hph* mutant/Δ*sav845*::*aphII*-mut-*loxP*. pKU470 was introduced into the strain for removal of a 0.42 Mb region covering from *sav434* to *sav845*.

For the SALD-3 mutant, the *aphII* gene of pLT152 was replaced with a *hph* gene harboring mut-*loxP* by *Spe*I digestion and ligation, resulting in pLT153. pLT153 was introduced into *S*. *avermitilis* KA-320 Δ*avaR3* mutant to yield *S*. *avermitilis* Δ*avaR3*/*sav845*::mut-*loxP*-*hph* mutant. Two regions (1,271,761–1,273,916 nt and 1,273,956–1,276,187 nt) were PCR-amplified by the primer pairs (sav1007-up*-*Fw/sav1007-up-Re and sav1007-dw*-*Fw/sav1007-dw*-*Re), and ligated with pKU451 to get pLT154. This plasmid was ligated with the *aphII* gene prepared previously to generate pLT155. A 6.0 kb *Hin*dIII fragment, recovered from pLT155, was cloned into pKU250 at the *Hin*dIII site to obtain pLT156. pLT156 was introduced to *S*. *avermitilis* Δ*avaR3*/*sav845*::mut-*loxP*-*hph* mutant, resulting in *S*. *avermitilis* Δ*avaR3*/*sav845*::mut-*loxP*-*hph* mutant/Δ*sav1007*::*aphII*-mut-*loxP*. pKU470 was introduced into the strain for removal of a 0.28 Mb region covering from *sav845* to *sav1007*.

For the SALD-4 mutant, the *aphII* gene of pLT156 was replaced with a *hph* gene harboring mut-*loxP* by *Spe*I digestion and ligation, resulting in pLT157. pLT157 was introduced into *S*. *avermitilis* KA-320 Δ*avaR3* mutant to yield *S*. *avermitilis* Δ*avaR3*/*sav1007*::mut-*loxP*-*hph* mutant. Two regions (1,593,533–1,595,311 nt and 1,595,580–1,597,661 nt) were PCR-amplified by the primer pairs (sav1286-up*-*Fw/sav1286-up-Re and sav1286-dw*-*Fw/sav1286-dw*-*Re), and ligated with pKU451 to get pLT158. This plasmid was ligated with the *aphII* gene prepared previously to generate pLT159. A 5.5 kb *Hin*dIII fragment, recovered from pLT159, was cloned into pKU250 at the *Hin*dIII site to obtain pLT160. pLT160 was introduced to *S*. *avermitilis* Δ*avaR3*/*sav1007*::mut-*loxP*-*hph* mutant, resulting in *S*. *avermitilis* Δ*avaR3*/*sav1007*::mut-*loxP*-*hph* mutant/Δ*sav1286*::*aphII*-mut-*loxP*. pKU470 was introduced into the strain for removal of a 0.32 Mb region covering from *sav1007* to *sav1286*.

The genotype of candidate strains for the desired large deletion mutation was confirmed by PCR analysis and DNA sequencing. The *S*. *avermitilis* KA-320 Δ*avaR3* lacking each region (*sav71*-*sav434*, *sav434*-*sav845*, *sav845-sav1007*, and *sav1007-sav1286*) was designated as SALD-1, SALD-2, SALD-3, and SALD-4.

### Genome sequencing and bioinformatics analyses

The genomic DNA of *S*. *avermitilis* KA-320 was prepared according to the previous procedures [24]. Sequence data of *S*. *avermitilis* KA-320 was obtained by assembling both data generated from the old-type DNA sequencer (MegaBACE 1000) and the next-generation DNA sequencer (Illumina). After assembling them, at least 21 contigs were generated. One contig (ca. 164 kb) contained the putative biosynthetic gene cluster for phthoxazolin A (a region around the gene cluster has been deposited in the DDBJ; accession number LC315614). Annotation of open reading frames (ORFs) and gene functions was performed manually by using the FramePlot 4.0beta program (http://nocardia.nih.go.jp/fp4), the 2ndFind program (http://biosyn.nih.go.jp/2ndfind/), the BLAST algorithm and the web-based PKS/NRPS analysis program (http://nrps.igs.umaryland.edu/nrps/).

### In-frame deletion of the *ptxA* gene in the *avaR3* mutant

Two 2.0 kb flanking regions of the *ptxA* gene were PCR-amplified by the primer pairs (ptxA-up-Fw/ptxA-up-Re and ptxA-dw-Re/ptxA-dw-Fw). These fragments were digested by *Hin*dIII and *Spe*I, and inserted into pKU451, resulting in pLT140. This plasmid was digested with *Spe*I and ligated together with a fragment containing a *hph* gene (PCR-amplified with pKU480 and the hph-Fw/hph-Re primer pair) to generate pLT141. A 6.2 kb *Hin*dIII fragment was recovered from pLT141, and cloned into the *Hin*dIII site of pKU250 to obtain pLT142. pLT142 was introduced into the *S*. *avermitilis* KA-320 Δ*avaR3* mutant by intergeneric conjugation [20], and the DNA region including the *ptxA* gene was replaced with the disrupted allele by homologous recombination. The genotype of candidate strains for the *ptxA* mutation was confirmed by PCR analysis and DNA sequencing. The *S*. *avermitilis* KA-320 *avaR3*/*ptxA* double mutant was abbreviated as Δ*avaR3* Δ*ptxA*.

### Complementation of the *avaR3*/*ptxA* double mutant

A 3.2 kb fragment containing the entire *ptxA* gene was PCR-amplified by using the primer pair ptxA-comp-Fw/ptxA-comp-Re, and then cloned to the *Bam*HI site of pLT101 [25] using GeneArt Seamless Cloning and Assembly Kit (Life Technologies). The resultant plasmid was introduced into the *avaR3*/*ptxA* double mutant by intergeneric conjugation and integration. Integration of the plasmid was confirmed by apramycin resistance and PCR analysis.

## Results

### Phthoxazolin A production in the *S*. *avermitilis* progeny

To identify a biosynthetic gene cluster for phthoxazolin A, *in silico* screening was performed with the public genome sequence of *S*. *avermitilis* using oxazolomycin biosynthetic genes as probes. However, we could not find any assembly line in the genome that was similar to that of the oxazolomycin biosynthesis. Moreover, the genome has no orthologue of OzmO (an NRPS enzyme), which is necessary for the biosynthesis of an oxazole ring moiety. It thus appeared that the public genome sequence contained no clues for identifying the phthoxazolin A biosynthetic gene cluster.

*S*. *avermitilis* K139 SUKA22 is genetically constructed by deleting the 1.5 Mb left region of the parental strain [22], and was found to lack the ability to produce phthoxazolin A (Fig 1B), suggesting that the deleted DNA region might contain the phthoxazolin A biosynthetic gene cluster. Thus, we generated several series of large-deletion mutants (SALD mutants) from the *S*. *avermitilis* KA320 Δ*avaR3* mutant [20], and evaluated phthoxazolin A production in these SALD mutants (S1 Fig). To our surprise, all the large-deletion mutants (SALD-1, SALD-2, SALD-3 and SALD-4 strains) still produced phthoxazolin A at a level comparable to the parental Δ*avaR3* mutant strain.

*S*. *avermitilis* K139 is one progeny of our wild-type strain (*S*. *avermitilis* KA-320, which is isogenic to MA-4680, ATCC 31267 and NRRL 8165), and K139 has been used in the genome sequencing project [18]. The strain K139 also did not produce phthoxazolin A (Fig 1B), suggesting that genetic differences may exist between *S*. *avermitilis* KA-320 and K139 to confer ability for the phthoxazolin A biosynthesis.

### Identification of the phthoxazolin A biosynthetic gene cluster

**Fig 2:**
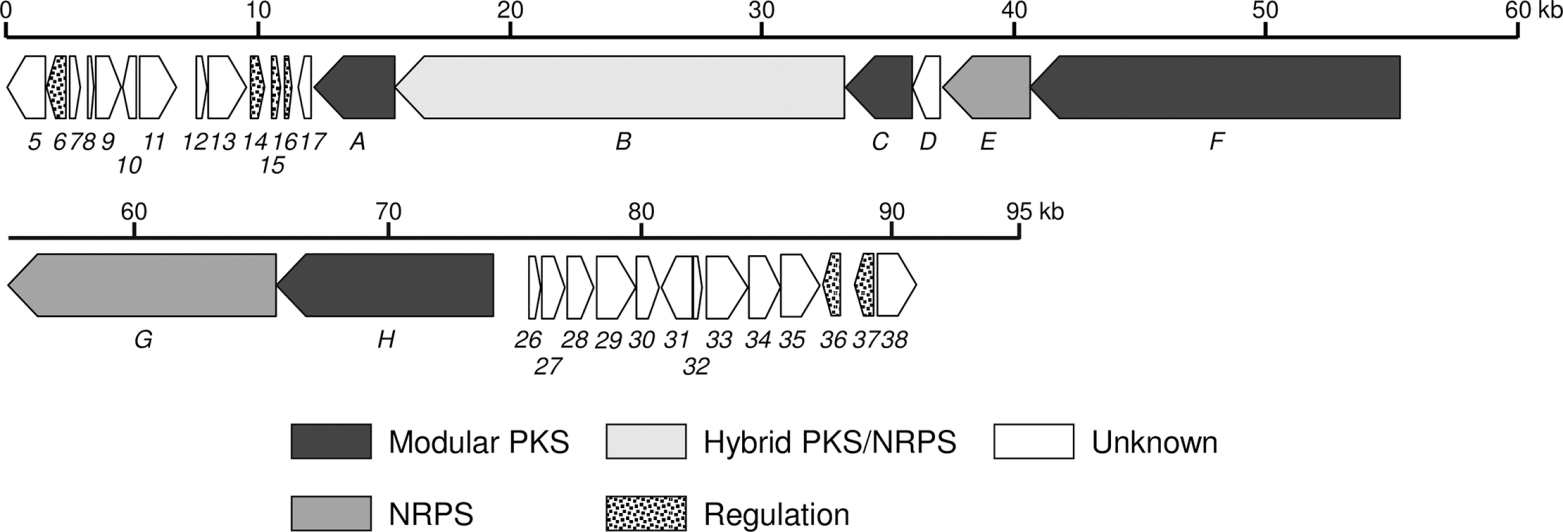
Genetic organization of the phthoxazolin A biosynthetic gene cluster. Arrows indicate the direction of transcription and relative gene size. ORFs predicted to participate in phthoxazolin A biosynthesis are shaded. The proposed functions of individual ORFs are indicated here and summarized in Table 1.

To reveal genetic differences between strains KA-320 and K139, we performed a contour-clamped homogeneous electrical field (CHEF) electrophoresis analysis of these chromosomes, and found that the genome size of strain KA-320 was about 800–1,000 kb larger than that of strain K139 and that the extra region of strain KA-320 was located at the right-hand region of the K139 genome. Based on these results, there was a strong possibility that the extra region of strain KA-320 encodes a biosynthetic gene cluster for phthoxazolin A. Thus, we sequenced the genome of strain KA-320 to find the extra region. By using the same approach as described above, we performed *in silico* screening with the genome sequencing of *S*. *avermitilis* KA-320, and found a possible phthoxazolin A biosynthetic gene cluster that spanned 99.9 kb (Fig 2). Annotation analysis of the sequence and comparison with genes in the public databases revealed 34 ORFs. The genetic organization and proposed functions are shown in Fig 2 and Table 1, respectively.

**Table 1:** Deduced functions of ORFs in the phthoxazolin A biosynthetic gene cluster.

Gene	Size^a^	Homolog^b^ and origin	Identity/ similarity (%)	Proposed function
*orf 5*	504	GlpK (WP_015654981), *Streptomyces davawensis* JCM 4913	91/95	Glycerol kinase
*orf 6*	255	ASC56_RS09905 (WP_055490882), *Streptomyces* sp.TP-A0356	92/96	IclR-family transcriptional regulator
*orf 7*	142	ADL25_RS11400 (WP_059127556), *Streptomyces* sp. NRRL F-5122	67/76	Histidine kinase
*orf 8*	80	IQ62_RS20385 (WP_037697207), *Streptomyces scabiei* NCPPB 4086	85/91	Hypothetical protein
*orf 9*	333	Ppk2 (WP_007385561), *Streptomyces sviceus* ATCC 29083	88/93	Polyphosphate kinase
*orf 10*	180	SAMN05216482_0059 (SEB58800), *Streptomyces* sp. PAN_FS17	78/87	Hypothetical protein
*orf 11*	481	G412_RS0110405 (WP_02881204), *Streptomyces flavidovirens* DSM 40150	96/98	Glyceraldehyde 3-phosphate dehydrogenase
*orf 12*	137	NF37_RS0107960 (WP_032755078), *Streptomyces alboviridis* NRRL B-1579	81/91	Integrase
*orf 13*	501	AWV61_RS50755(WP_060880896), *Streptomyces scabiei* 95–18	83/85	Transposase
*orf 14*	188	AVL59_RS26005 (WP_067308799), *Sreptomyces griseochromogenes* ATCC 14511	81/86	Two-component system sensor kinase
*orf 15*	113	CCN44_RS40620 (WP_086704188), *Streptomyces tricolor* NRRL B-16925	86/86	Two-component system response regulator
*orf16*	94	IG08_RS0113085 (WP_030600335), *Streptomyces fulvoviolaceus* NRRL B-2870	87/88	LuxR-family transcriptional regulator
*orf 17*	169	BIV24_RS13170 (WP_071366454), *Streptomyces* sp. MUSC 93	74/82	Polyketide cyclase
*ptxA*	1065	OzmM (ABS90474), *Streptomyces albus* JA3453	57/68	Acetyl transferase
*ptxB*	5939	OzmH (ABS90470), *S*. *albus* JA3453	53/62	Hybrid NRPS-PKS
*ptxC*	877	OzmQ (ABS90478), *S*. *albus* JA3453	67/76	Type I PKS
*ptxD*	362	OzmP (ABS90477), *S*. *albus* JA3453	77/88	Hypothetical protein
*ptxE*	1154	OzmO (ABS90476), *S*. *albus* JA3453	53/62	NRPS
*ptxF*	4885	OzmN (ABS90475), *S*. *albus* JA3453	51/60	Type I PKS
*ptxG*	3542	NRPS (OMI35273), *Streptomyces sparsogenes* DSM 40356	65/74	NRPS
*ptxH*	2860	PKS 1–1 (ADI03434), *Streptomyces bingchenggensis* BCW-1	63/72	Type I PKS
*orf 26*	155	SibV (ACN39745), *Streptosporangium sibiricum* DSM 44039	66/77	Dioxygenase
*orf 27*	306	BZL62_RS04865 (WP_086716558), *Streptomyces angustmyceticus* NRRL B-2347	68/78	Hypothetical protein
*orf 28*	347	AOK13_RS10670 (WP_055559528), *Streptomyces luridiscabiei* NRRL B-24455	81/87	IMP dehydrogenase
*orf 29*	517	BZL62_RS04825 (WP_086716551), *Streptomyces angustmyceticus* NRRL B-2347	79/86	Acetolactate synthase
*orf 30*	295	SAMN05444920_109123 (SEG95653), *Nonomuraea solani* CGMCC 4.7037	55/70	Hydroxyacid dehydrogenase
*orf 31*	410	BR98_RS37570 (WP_083976095), *Kitasatospora azatica* KCTC 9699	70/82	Cytochrome P450
*orf 32*	71	WT80_RS35315 (WP_081087741) *Burkholderia stagnalis* MSMB774WGS	43/56	unknown
*orf 33*	526	KCH_RS21250 (WP_084223811), *Kitasatospora cheerisanensis* KCTC 2395	66/77	Fatty acid Co A ligase
*orf 34*	409	SAMN05216533_5065 (SEF00159), *Streptomyces* sp. Ag109_O5-10	85/92	5-Aminolevulinate synthase
*orf 35*	509	Ann2 (AGY30678), *Streptomyces calvus* ATCC 13382	67/81	5-Aminolevulinate synthase
*orf 36*	225	ColR1 (AIL50186), *Streptomyces aureus* SOK1/5-04	50/62	LuxR-family transcriptional regulator
*orf 37*	248	IF01_RS0119920 (WP_051755722), *Streptomyces purpeofuscus* NRRL B-1817	75/83	TetR-family transcriptional regulator
*orf 38*	516	OO66_RS31780 (WP_051763676), *Streptomyces virginiae* NRRL B-8091	74/83	Multidrug MFS (major facilitator superfamily) transporter

^a^ Numbers refer to amino acid residues.

^b^ Parenthetical codes are National Center for Biotechnology Information accession numbers.

**Fig 3:**
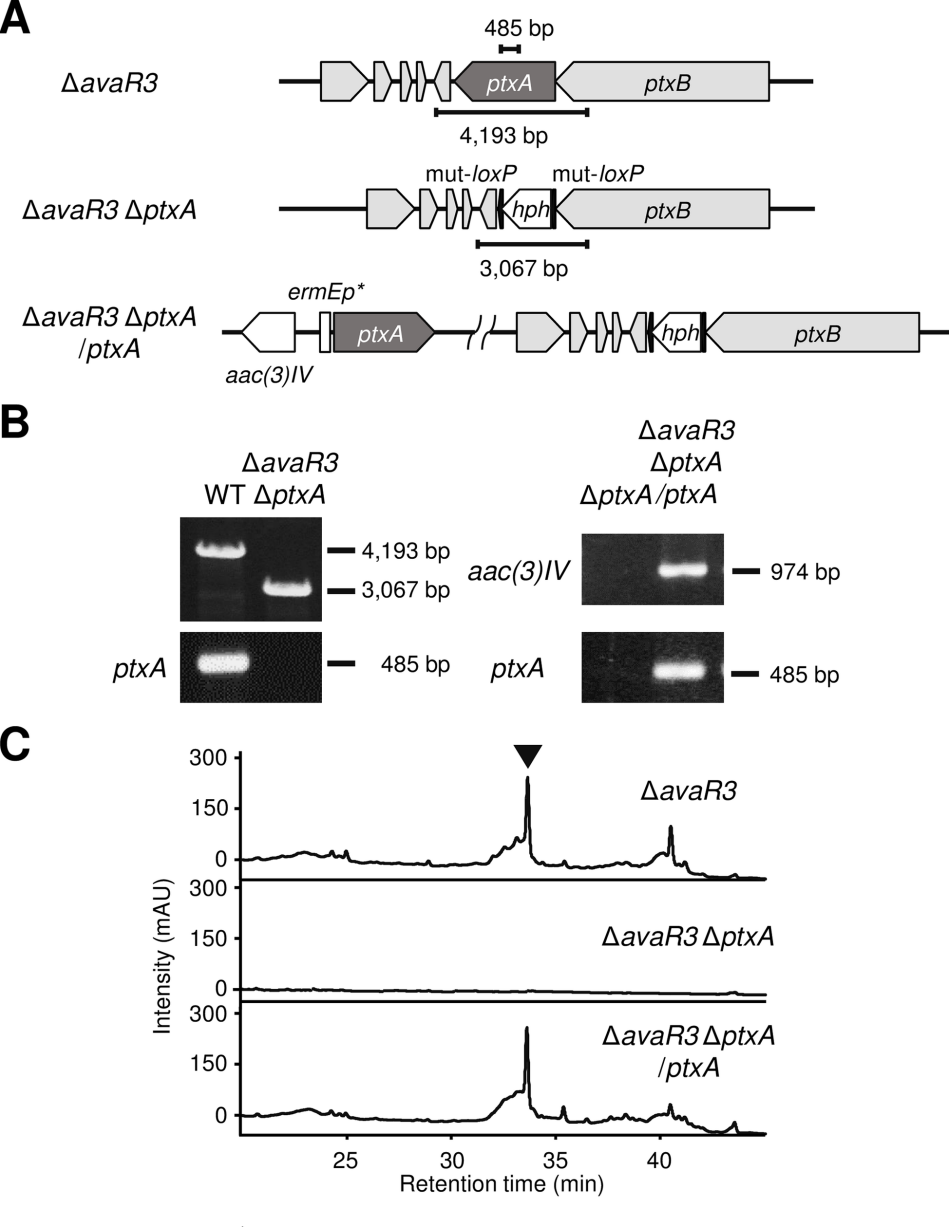
Phthoxazolin A production in the *avaR3*/*ptxA* double mutant. (A) Schematic representation of the strategy for the *ptxA* gene disruption. Δ*avaR3*, *avaR3* mutant; Δ*avaR3* Δ*ptxA*, *avaR3*/*ptxA* double mutant; Δ*avaR3* Δ*ptxA/ptxA*, *ptxA*-complemented *avaR3*/*ptxA* double mutant. (B) PCR analysis to confirm gene-disruption of the *ptxA* gene and its complementation. With the primer pair ptzA-tFw/ptzA-tRe, a fragment (4,193 bp) containing an intact *ptxA* gene or a fragment (3,067 bp) containing the mut-*loxP*-*hph*-mut-*loxP* was amplified with PCR. An intact *ptxA* gene (485 bp) was detected by using the primer pair ptzA-Fw/ptzA-Re. An internal region of *aac(3)IV* gene (974 bp) was amplified using the primer pair apr-Fw/apr-Re. (C) HPLC chromatograms of MeOH extracts from the *avaR3/ptxA* double mutant. mAU, milliabsorbance units at 275 nm. Phthoxazolin A is indicated by an inverted triangle.

Three PKS genes (*ptxC*, *ptxF*, and *ptxH*), one hybrid NRPS/PKS gene (*ptxB*), and two NRPS genes (*ptxE* and *ptxG*) were identified in the cluster, together with *ptxA*, which encoded a putative discrete AT enzyme. To investigate the involvement of PtxA in the biosynthesis of phthoxazolin A, we disrupted the *ptxA* gene by deleting 1,032 amino acids in the Δ*avaR3* genetic background. The double mutant (Δ*avaR3* Δ*ptxA*) strain was unable to produce phthoxazolin A (Fig 3) and the *ptxA*-complemented *avaR3*/*ptxA* double mutant (Δ*avaR3* Δ*ptxA*/*ptxA*) produced phthoxazolin A to the level in the parental strain (Δ*avaR3*) (Fig 3), indicating that the discrete AT (PtxA) is essential for the phthoxazolin A biosynthesis. This result also suggested that the PKS genes and the hybrid NRPS/PKS gene, which require enzymatic activity of PtxA, are involved in the biosynthesis of phthoxazolin A (see next section).

### A gene encoding a discrete AT

The PtxA protein contains two tandem AT domains together with an oxidoreductase domain, and should function as a discrete AT enzyme in the *trans*-AT type I PKS gene cluster. In the *ptx* gene cluster, three PKS genes (*ptxC*, *ptxF*, and *ptxH*) and a hybrid NRPS/PKS gene (*ptxB*) are embedded to encode 10 PKS modules (modules 1 to 5 and 7 to 11). The PKS modules encoded by *ptxB*, *ptxC*, *ptxF*, and *ptxH* all lack cognate AT domains, implying that the PtxA protein provides the missing AT activity by acting *in trans* for these PKS modules. The oxazolomycin pathway has also been established to have the *trans*-AT type I PKS system with a discrete AT enzyme (OzmM containing the tandem AT domains) [12]. Phylogenetic tree analysis of the AT domains from PtxA (PtxA-AT1 and PtxA-AT2) with other tandem-type discrete AT enzymes revealed that PtxA-AT1 and Ozm-AT1 are positioned in a different clade from PtxA-AT2, Ozm-AT2 and KirC1-AT2 (S2 Fig). Zhao *et al*. [12] established that OzmM-AT2 is involved in the oxazolomycin biosynthesis, whereas OzmM-AT1 is dispensable. Moreover, KirC1-AT2 loads malonyl-CoA extender units to the ACPs in the kirromycin biosynthesis [26]. With the observation that PtxA-AT1 seems inactive due to the replacement of important amino acid residues [27] (substitution of Glu^63^ and His^91^ with His and Ser, respectively), much as in the cases of OzmM-AT1 and KirC1-AT1, it can be concluded that PtxA-AT2 supplies malonyl-CoA units to the Ptx PKS modules.

### Genes involved in the polyketide assembly

Ten PKS modules were identified in the *ptx* gene cluster, encoded by *ptxB*, *ptxC*, *ptxF*, and *ptxH*. PtxC and PtxF closely resembled OzmQ (67% identity) and OzmN (51% identity), respectively. Both OzmQ and OzmN have PKS modules 2, 3, 4, and 5 in the oxazolomycin assembly line, and render a triene moiety in the structure of oxazolomycin [12]. PKS modules 4 and 5 of PtxB contain two methyltransferase (MT) domains for the addition of methyl groups at C-2 and C-4 of phthoxazolin A.

With respect to the KS domains in the *ptx* cluster, analysis of the conserved active site (catalytic triad of Cys-His-His) [28] revealed that two KS domains (KS9 and KS11) contain a mutation in the conserved motif (S3 Fig), suggesting that they should be inactive in the polyketide assembly line; these domains were called KS^0^ domains [14]. Regarding the ACP domains, the Ser residue in the signature motif plays a crucial role as the 4’-phosphopantetheine attachment site [29], but the first ACP domain (ACP9a) of module 9 lacks the Ser residue, while other ACP domains in the Ptx PKS modules retain the conserved Ser residue (S4 Fig), indicating that the PKS module 9 employs two types of ACPs (inactive-type and active-type). Along with the ketoreductase (KR) domains, seven KR domains are predicted in the PKS modules, all of which show a characteristic Rossmann fold for NADP(H)-binding [30] (S5 Fig). In addition, all the KR domains except KR4 and KR5 contain a typical catalytic triad (Ser-Tyr-Asn) [30], while KR4 and KR5 have different sequences (Ser-Tyr-Cys for KR4 and Ser-Tyr-Ser for KR5). Because these minor modifications in the catalytic triad have also been identified in other *trans*-AT type I PKSs, such as those in OocL and OocR in the oocydin A biosynthesis [31], all the *ptx* KR domains seem to be active in the PKS assembly line. Regarding the DH domains, all six DH domains possess the conserved His residue in the signature motif HXXXGXXXXP [32] (S6 Fig), indicating that the dehydratase (DH) domains are active, and are likely responsible for the formation of a double bond. Taking these results together, the first 5 PKS modules (modules 1 to 5) appear to be responsible for synthesizing the polyketide moiety of phthoxazolin A, whereas the remaining 5 PKS modules (modules 7, 8, 9, 10, and 11) might be capable of synthesizing an additional triene structure (as discussed later).

### Genes involved in the formation of an oxazole ring

The *ptxE* gene encodes a single module of NRPS comprising a formylation (F) domain, an A domain, and a PCP domain, which probably serve as loading modules to incorporate an initial amino acid into the assembly line. Because the PtxE-A domain is predicted to be a glycine-specific A domain based on bioinformatic analysis using the AntiSMASH database [33], glycine can be considered to be activated by the PtxE-A domain and loaded onto the PtxE-P domain as a glycyl-*S*-PCP. The presence of an F domain in the loading module has sometimes been identified in other biosynthetic machineries, such as those for the biosynthesis of gramicidin [34], rhizopodin [35], oxazolomycin [12], and conglobatin [11], and the PtxE-F domain can be predicted to catalyze formylation of glycyl-*S*-PCP to generate formyl-glycyl-*S*-PCP, from the analogy of the F domain of LgrA1 in the gramicidin biosynthesis.

The downstream region of the *ptxE* gene includes the *ptxD* gene, which encodes a protein homologous to OzmP (in oxazolomycin biosynthesis) and CongE (in conglobatin biosynthesis). The gene arrangement (*ptxD*-*ptxE*) is identical to those in the biosynthetic gene clusters of oxazolomycin (*ozmO*-*ozmP*) and conglobatin (*congA*-*congE*). Moreover, these three proteins (PtxD, CongE, and OzmP) have an ATP-pyrophosphatase domain that includes the signature motif SGGKDS in the N-termini for ATP binding [36]. Because CongE has been proposed to activate the amide oxygen by adenyltransfer from ATP with the release of pyrophosphate for formation of the oxazole ring moiety [11], PtxD is likely to be responsible for conversion of the formyl-glycyl intermediate into the oxazole ring by cyclodehydration.

### Genes putatively involved in the nonribosomal peptide assembly

The assembly line in the *ptx* gene cluster possesses four NRPS modules, including a loading module encoded by *ptxE*. NRPS module 6 is a part of the PKS/NRPS hybrid protein PtxB, and NRPS modules 12 and 13 are encompassed in the PtxG protein. The specificity-conferring codes of the A domains indicated that amino acids activated by PtxB-A6, PtxG-A12 and PtxG-A13 are glycine, serine and tyrosine, respectively. The glycine residue recognized by PtxB-A6 would be incorporated into the structure of phthoxazolin A as a part of a carbamoyl moiety. The terminal module 13 of the PtxG protein includes an *N*-MT domain and a cytochrome P450 domain. The MT domain probably modifies the Tyr residue incorporated by PtxG-A13. Because the NRPS module 6 is likely to be a final module in the phthoxazolin A assembly line, it is difficult to predict the involvement of the NRPS modules 12 and 13 in phthoxazolin A biosynthesis at present.

### Proposed model for phthoxazolin A biosynthesis

**Fig 4:**
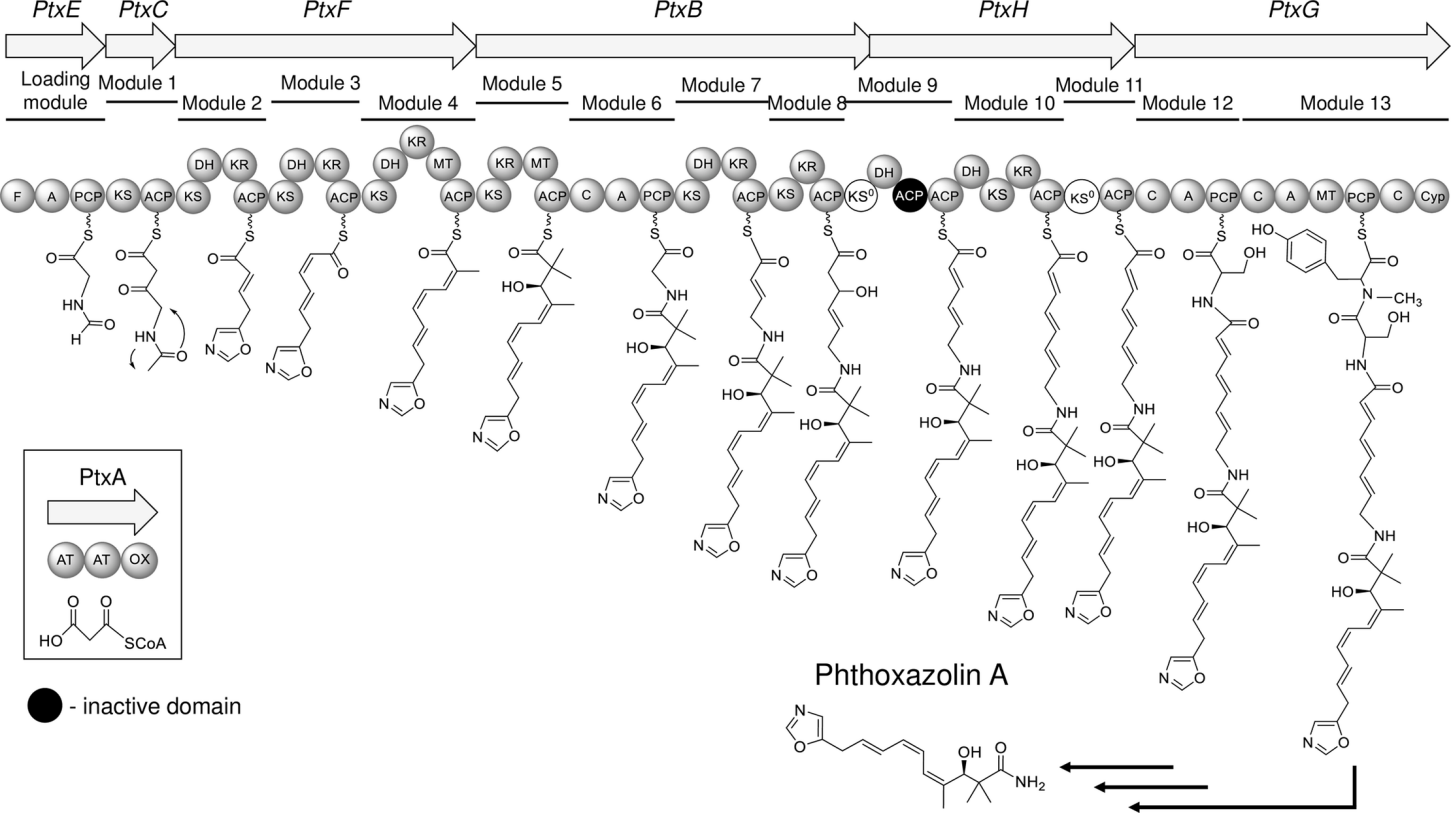
Proposed model for phthoxazolin A biosynthesis. A, adenylation; ACP, acyl carrier protein; AT, acyltransferase; C, condensation; Cyp, cytochrome P450; DH, dehydratase; F, formylation; KS, ketosynthase; KS^0^, KS lacking His in the HTGTG motif; KR, ketoreductase; MT, methyltransferase; PCP, peptidyl carrier protein. The presumed inactive ACP domain of module 9 is shaded in black.

The *in silico* analyses of the Ptx proteins described above allowed us to propose a model for the phthoxazolin A biosynthesis (Fig 4). Phthoxazolin A biosynthesis starts with activation of glycine and the tethering to the PCP domain of PtxE, followed by formylation by the F domain to generate a formyl-glycyl-*S*-PCP. PKS module 1 of PtxC, representing a minimal module of the *trans*-AT type I PKS systems, incorporates a malonyl-CoA into the formyl-glycyl intermediate. The cyclodehydration by PtxD on the intermediate would occur after the condensation between the formyl-glycyl-*S*-PCP and the malonyl-CoA, although the timing of cyclodehydration remains unclear. Subsequently, four malonyl-CoA units and three methyl groups are incorporated by four PKS modules (modules 2 to 5) of PtxF and PtxB. The KR-DH domain pair in the modules 2, 3 and 4 provides three conjugated double bonds (C8-C9, C6-C7 and C4-C5), and generates the *trans*, *cis*, and *cis* configuration of double bonds, respectively. KR5 of PKS module 5 could be classified as a KR domain found in the partially reducing PKS [37], and introduces a hydroxyl group in the *R* configuration at C-3 of phthoxazolin A. NRPS module 6 activates a glycine residue, and loads it onto the polyketide intermediate to generate a carbamoyl moiety. The remaining five PKS modules could produce a triene moiety, and two NRPS modules could incorporate two additional amino acids (Ser and *N*-Tyr) into the polyketide assembly line. However, considering the structure of phthoxazolin A, these additional substructures are unnecessary for the biosynthesis of phthoxazolin A. The thioesterase (TE) domain usually catalyzes the release of a completed chain of PKS/NRPS products from the assembly line [38], but no such TE domain was found in the *ptx* assembly line. Finally, the product generated by the *ptx* chain assembly should be processed by some enzymatic reactions to complete phthoxazolin A biosynthesis. To date, however, no clear candidate enzyme has been found in the *ptx* gene cluster or its adjacent regions.

## Discussion

The oxazole-containing polyketides have the distinct feature of an oxazole-moiety linked with a polyketide structure or a hybrid peptide-polyketide structure, and bestow remarkable biological activities such as antibacterial, antitumor and herbicidal properties. Only a few biosynthetic gene clusters of oxazole-containing polyketides have been identified [10–12,35,39] and their biosynthetic mechanisms have provided us valuable genetic information of the complicated pathways involved in heterocycles formation and sequential extensions by PKSs or PKS/NRPSs. Recently, we identified phthoxazolin A, which is a polyketide compound containing a 5-substituted oxazole ring and a triene moiety, as a cryptic metabolite of the original avermectin producer *S*. *avermitilis* KA-320 [18]. In the present study, we have shown that strain KA-320 harbors an extra genomic region, which is absent in the publicly available genome of strain K139, and that a biosynthetic gene cluster for phthoxazolin A is laid on the extra region of strain KA-320. The biosynthetic gene cluster could be classified into a growing number of *trans*-AT type I PKS systems that utilize a discrete AT enzyme to supply a malonyl-CoA unit into all the PKS modules as opposed to the *cis*-acting integrated AT domains of canonical PKSs [13,14].

The structure of phthoxazolin A resembles the partial structure of oxazolomycin. Comparison of the functional domains of the biosynthetic gene clusters revealed a virtually complete architectural identity with the corresponding portion of the *ptx* and *ozm* PKS-NRPS clusters over the first eight modules, with the exception of a dispensable module (PKS module 7) for the phthoxazolin A biosynthesis. The Ptx proteins are 51%-71% identical to their orthologues in the *ozm* cluster. Similar to the oxazolomycin biosynthetic pathway, the phthoxazolin A biosynthesis is initiated by formylation of the glycine residue, followed by extension by a malonyl-CoA unit and cyclodehydration to generate an oxazole ring moiety. The biosynthetic maturation of phthoxazolin A should require additional extension by three malonyl-CoA units to yield a triene moiety and termination by glycine incorporation to generate a carbamoyl moiety. The geometries of the conjugated triene moiety of phthoxazolin A are assigned as 4*Z*,6*Z*,8*E*. A PKS module encompassing the pair of a B-type KR domain and a DH domain normally biosynthesize polyketide chain elongation intermediates with a *trans* (*E*) double bond [30]. Sequence analysis of KR domains in the *ptx* cluster demonstrated that KR2, KR3, and KR4 contain the stereochemistry signature “LDD” motif for B-type KR domains [40, 41], although their motifs vary among the individual KR domains. These analyses indicated that the polyketide intermediate of phthoxazolin A is expected to have three *trans* conjugated double bonds, which are different from those of phthoxazolin A. Whether the change of stereochemistry in the biosynthetic process requires an enzymatic reaction remains obscure. However, this contradictory feature for the formation of conjugated double bonds has also been observed in the biosynthetic pathways of oxazolomycin and chivosazol [12,39].

The *ptx* gene cluster encodes a series of modules indispensable (from the loading module to NRPS module 6) for the phthoxazolin A biosynthesis, but also includes additional functional modules (PKS module 7 to NRPS module 13) for the biosynthesis of an unidentified compound, which might be a precursor of phthoxazolin A. In particular, PKS modules 8 and 9 include the interesting feature of a non-elongating KS (KS^0^) domain. These modules are composed of KS-KR-ACP-KS^0^ domains followed by DH-ACP domains, and are grouped as type A bimodules for dehydration [14,42] In addition, the domain pair of KS^0^-ACP as a non-elongating module is also embedded in PKS module 11. Both the type A bimodule for dehydration and the domain pair of KS^0^-ACP are ubiquitously found in the *trans*-AT type I PKS system [14]. The remaining modules (PKS modules 10 and 11, and NRPS modules 12 and 13) also might be responsible for a polyketide elongation and an incorporation of amino acids, as well as those of the oxazolomycin biosynthesis, suggesting that the *ptx* gene cluster would produce a larger compound than that of phthoxazolin A; namely, phthoxazolin A is a cleaved compound of the ancestral compound. HPLC analysis of the *avaR3*/*ptxA* double mutant demonstrated that, in addition to phthoxazolin A, the strain lost production of several more compounds that are present in the parental strain (Fig 3C): any of the compounds can be the ancestral compounds synthesized by the *ptx* gene cluster. In the pederin biosynthetic pathway, a putative FAD-dependent monooxygenase (PedG) has been proposed to oxidatively cleave the final product generated by the whole assembly line and produce a pederin precursor [43]. On the other hand, the *ptx* gene cluster and its flanking regions have no genes encoding an FAD-dependent monooxygenase, suggesting that another cleavage mechanism might be applied to the synthesis of phthoxazolin A.

In conclusion, we have shown that an extra genomic region of the original avermectin producer has a unique *trans*-AT type I PKS system for the biosynthesis of phthoxazolin A. These findings could provide further insight into the diversity of *trans*-AT type I PKS systems, which are widely distributed in bacteria [6,14]. Moreover, our finding of additional PKS/NRPS modules in the *ptx* assembly line suggests that *S*. *avermitilis* is capable of producing a larger intermediate than phthoxazolin A, and that the PKS/NRPS machinery incorporates a new cleavage system. Further understanding of the phthoxazolin A biosynthetic pathway should provide interesting perspectives into the engineering of polyketide/non-ribosomal peptide biosynthetic pathways.

## Supporting information

S1 Fig Phthoxazolin A production in the SALD mutants. (A) Schematic representation for the construction of *Streptomyces avermitilis* large-deletion (SALD) mutant strains. The details of the construction are described in the Materials and Methods. Gray solid line represents the extra genomic region of *S*. *avermitilis* KA-320, and gray dashed lines represent the deleted DNA region in the genome. KA-320, *S*. *avermitilis* KA-320; K139, *S*. *avermitilis* K139; SUKA22, *S*. *avermitilis* SUKA22 (K139 as a genetic background). (B) HPLC chromatograms of MeOH extracts from *S*. *avermitilis* SALD mutants. KA-320/Δ*avaR3*, *S*. *avermitilis* Δ*avaR3* (KA-320 as genetic background). mAU, milliabsorbance units at 275 nm. Phthoxazolin A is indicated by an inverted triangle.
(PDF)

S2 Fig Phylogenetic analysis of AT domains of the *trans*-AT PKS system. BaeC (CAG23950), BaeD (CAG23951), and BaeE (CAG23952) from *Bacillus amyloliquefaciens* FZB42; BatH (ADD82949) and BatJ (ADD82951) from *Pseudomonas fluorescens* BCCM_ID9359; BryP (ABK51299) from *Candidatus endobugula sertula*; DifA (CAG23974) from *Bacillus amyloliquefaciens* FZB42; DszD (AAY32968) from *Sorangium cellulosum* So ce12; ElsA (WP_012792904) and ElsB (WP_012792903) from *Chitinophaga pinensis* DSM 2588; KirC1 (CAN89639) from *Streptomyces collinus* Tu 365; LnmG (AAN85520) from *Streptomyces atroolivaceus*; LkcD (BAC76473) from *Streptomyces rochei*; MmpC (AAM12912) from *P*. *fluorescens* NCIMB 10586; OzmM (ABS90474) from *S*. *albus* JA3453 and OzmM (ADI12766) from *Streptomyces bingchenggensis* BCW-1; PedC (AAS47559) and PedD (AAS47563) from symbiont bacterium of *Paederus fuscipes*; PsyH (ADA82589) from an unculturable symbiont of sponge *Psammocinia* aff. *Bulbosa*; RhiG (CAL69887) from *Burkholderia rhizoxina*; SorO (ADN68489) from *S*. *cellulosum* So ce12; VirI (BAF50719) from *Streptomyces virginiae*; FabD (CAA77658) form *E*. *coli*; and FabD SAV5788 (BAC73500) from *S*. *avermitilis*.
(PDF)

S3 Fig Sequence alignment of the conserved motifs in the KS domain core regions from *ptx* PKSs. The conserved catalytic triad of C-H-H is marked with an asterisk. The numbers indicate amino acid positions within each domain.
(PDF)

S4 Fig Sequence alignment of the conserved motif in the ACP/PCP domain core regions from *ptx* PKSs and NRPSs. The Ser residue functioning as the phosphopantetheine-binding site is marked with an asterisk. The numbers indicate amino acid positions within each domain.
(PDF)

S5 Fig Sequence alignment of the conserved motifs in the KR domain core regions from *ptx* PKSs. The conserved catalytic residues are marked with asterisks. The core region for the NADP(H)-binding motif is underlined. The numbers indicate amino acid positions within each domain. The “LDD” motif for B-type KR domains is shown in a box.
(PDF)

S6 Fig Sequence alignment of the conserved motif in the DH domain core regions from *ptx* PKSs. The proposed active catalytic His residue is marked with an asterisk. The numbers indicate amino acid positions within each domain.
(PDF)

S1 Table Oligonucleotides used in this study. (PDF)
