# Supplementary material for: Characterization of the biosynthetic gene cluster for cryptic phthoxazolin A in Streptomyces avermitilis
Source: PLoS One. 2018 Jan 11;13(1):e0190973. doi: 10.1371/journal.pone.0190973 (PMC5764310; doi:10.1371/journal.pone.0190973)
Supplement: S1 Fig — (A) Schematic representation for the construction of Streptomyces avermitilis large-deletion (SALD) mutant strains. The details of the construction are described in the Materials and Methods. Gray solid line represents the extra genomic region of S. avermitilis KA-320, and gray dashed lines represent the deleted DNA region in the genome. KA-320, S. avermitilis KA-320; K139, S. avermitilis K139; SUKA22, S. avermitilis SUKA22 (K139 as a genetic background). (B) HPLC chromatograms of MeOH extracts from S. avermitilis SALD mutants. KA-320/ΔavaR3, S. avermitilis ΔavaR3 (KA-320 as genetic background). mAU, milliabsorbance units at 275 nm. Phthoxazolin A is indicated by an inverted triangle. (PDF) [file pone.0190973.s001.pdf]

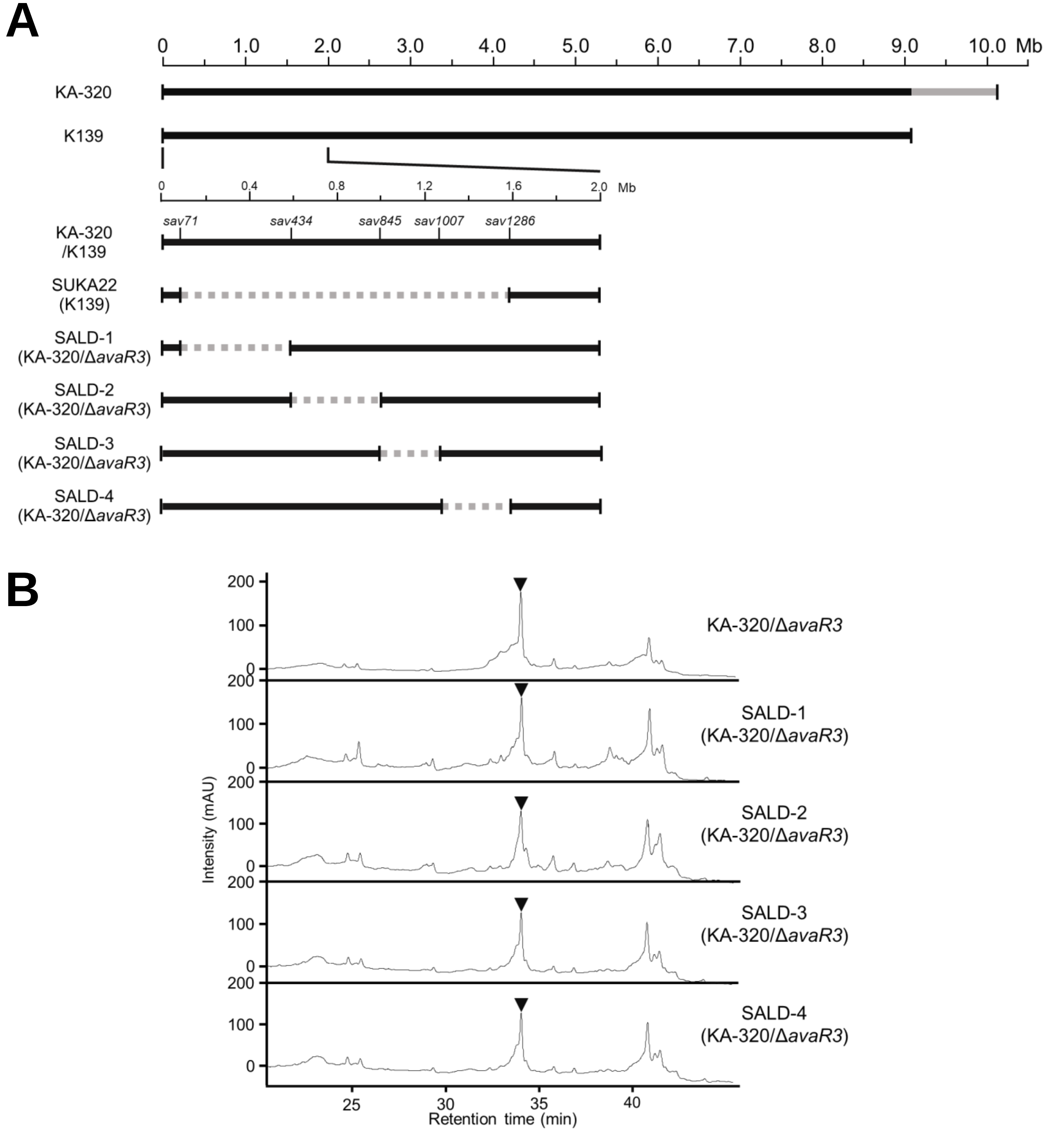

S1 Fig. Phthoxazolin A production in the SALD mutants. (A) Schematic representation for the construction of *Streptomyces avermitilis* large-deletion (SALD) mutant strains. The details of the construction are described in the Material and Methods. Gray solid line represents the extra genomic region of *S. avermitilis* KA-320, and gray dashed lines represent the deleted DNA region in the genome. KA-320, *S. avermitilis* KA-320; K139, *S. avermitilis* K139; SUKA22, *S. avermitilis* SUKA22 (K139 as a genetic background). (B) HPLC chromatograms of MeOH extracts from *S. avermitilis* SALD mutants. KA-320/Δ*avaR3*, *S. avermitilis* Δ*avaR3* (KA-320 as genetic background). mAU, milliabsorbance units at 275 nm. Phthoxazolin A is indicated by an inverted triangle.
