# Supplementary material for: Characterization of the biosynthetic gene cluster for cryptic phthoxazolin A in Streptomyces avermitilis
Source: PLoS One. 2018 Jan 11;13(1):e0190973. doi: 10.1371/journal.pone.0190973 (PMC5764310; doi:10.1371/journal.pone.0190973)
Supplement: S2 Fig — BaeC (CAG23950), BaeD (CAG23951), and BaeE (CAG23952) from Bacillus amyloliquefaciens FZB42; BatH (ADD82949) and BatJ (ADD82951) from Pseudomonas fluorescens BCCM_ID9359; BryP (ABK51299) from Candidatus endobugula sertula; DifA (CAG23974) from Bacillus amyloliquefaciens FZB42; DszD (AAY32968) from Sorangium cellulosum So ce12; ElsA (WP_012792904) and ElsB (WP_012792903) from Chitinophaga pinensis DSM 2588; KirC1 (CAN89639) from Streptomyces collinus Tu 365; LnmG (AAN85520) from Streptomyces atroolivaceus; LkcD (BAC76473) from Streptomyces rochei; MmpC (AAM12912) from P. fluorescens NCIMB 10586; OzmM (ABS90474) from S. albus JA3453 and OzmM (ADI12766) from Streptomyces bingchenggensis BCW-1; PedC (AAS47559) and PedD (AAS47563) from symbiont bacterium of Paederus fuscipes; PsyH (ADA82589) from an unculturable symbiont of sponge Psammocinia aff. Bulbosa; RhiG (CAL69887) from Burkholderia rhizoxina; SorO (ADN68489) from S. cellulosum So ce12; VirI (BAF50719) from Streptomyces virginiae; FabD (CAA77658) form E. coli; and FabD SAV5788 (BAC73500) from S. avermitilis. (PDF) [file pone.0190973.s002.pdf]

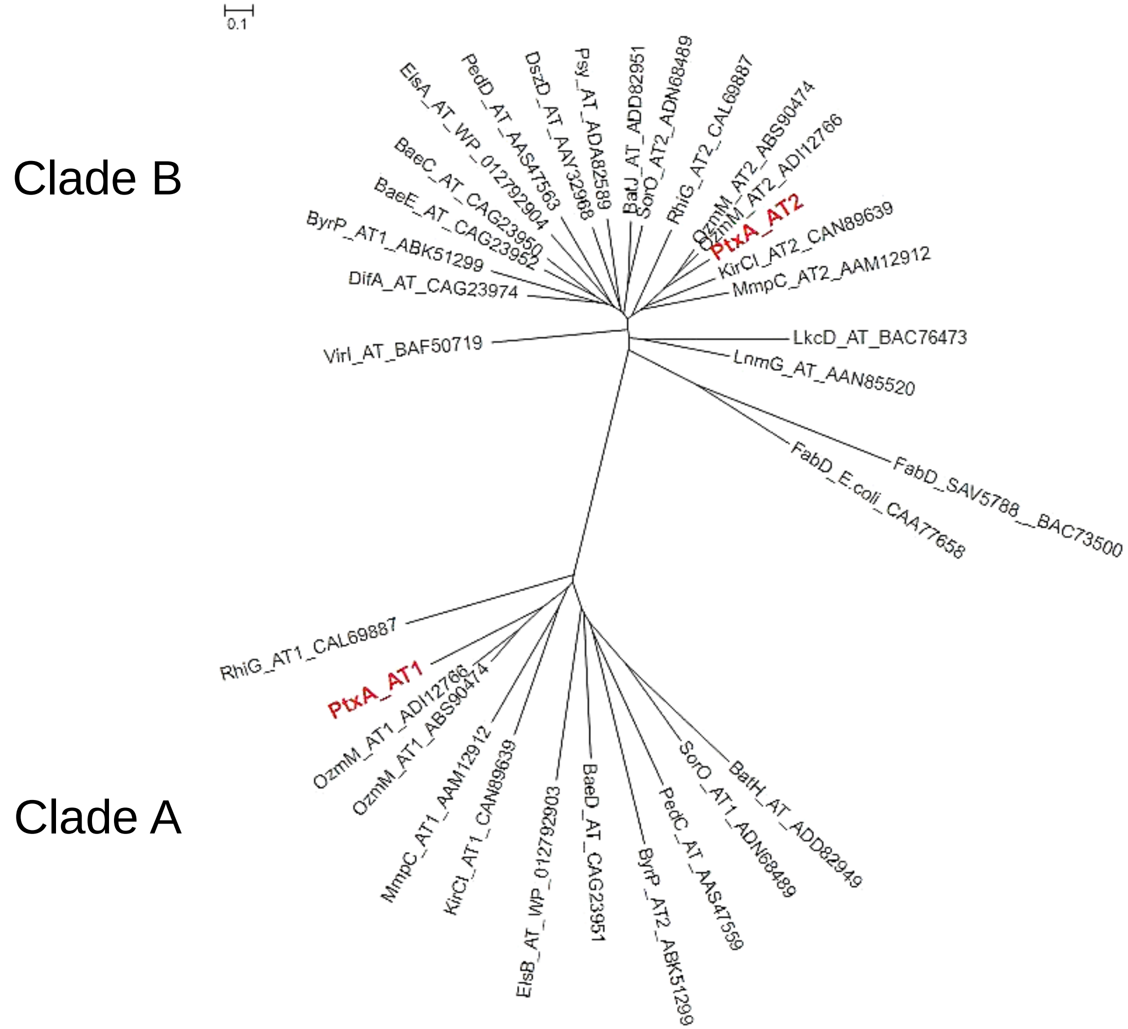

S2 Fig. Phylogenetic analysis of AT domains of the *trans-AT* PKS system. BaeC (CAG23950), BaeD (CAG23951), and BaeE (CAG23952) from *Bacillus amyloliquefaciens* FZB42; BatH (ADD82949) and BatJ (ADD82951) from *Pseudomonas fluorescens* BCCM_ID9359; BryP (ABK51299) from *Candidatus endobugula sertula*; DifA (CAG23974) from *Bacillus amyloliquefaciens* FZB42; DszD (AAY32968) from *Sorangium cellulosum* So ce12; ElsA (WP_012792904) and ElsB (WP_012792903) from *Chitinophaga pinensis* DSM 2588; KirC1 (CAN89639) from *Streptomyces collinus* Tu 365; LnmG (AAN85520) from *Streptomyces atroolivaceus*; LkcD (BAC76473) from *Streptomyces rochei*; MmpC (AAM12912) from *P. fluorescens* NCIMB 10586; OzmM (ABS90474) from *S. albus* JA3453 and OzmM (ADI12766) from *Streptomyces bingchenggensis* BCW-1; PedC (AAS47559) and PedD (AAS47563) from symbiont bacterium of *Paederus fuscipes*; PsyH (ADA82589) from an unculturable symbiont of sponge *Psammocinia* aff. *Bulbosa*; RhiG (CAL69887) from *Burkholderia rhizoxina*; SorO (ADN68489) from *S. cellulosum* So ce12; VirI (BAF50719) from *Streptomyces virginiae*; FabD (CAA77658) form *E. coli*; and FabD SAV5788 (BAC73500) from *S. avermitilis*.
