# Supplementary material for: Characterization of the biosynthetic gene cluster for cryptic phthoxazolin A in Streptomyces avermitilis
Source: PLoS One. 2018 Jan 11;13(1):e0190973. doi: 10.1371/journal.pone.0190973 (PMC5764310; doi:10.1371/journal.pone.0190973)
Supplement: S4 Fig — The Ser residue functioning as the phosphopantetheine-binding site is marked with an asterisk. The numbers indicate amino acid positions within each domain. (PDF) [file pone.0190973.s004.pdf]

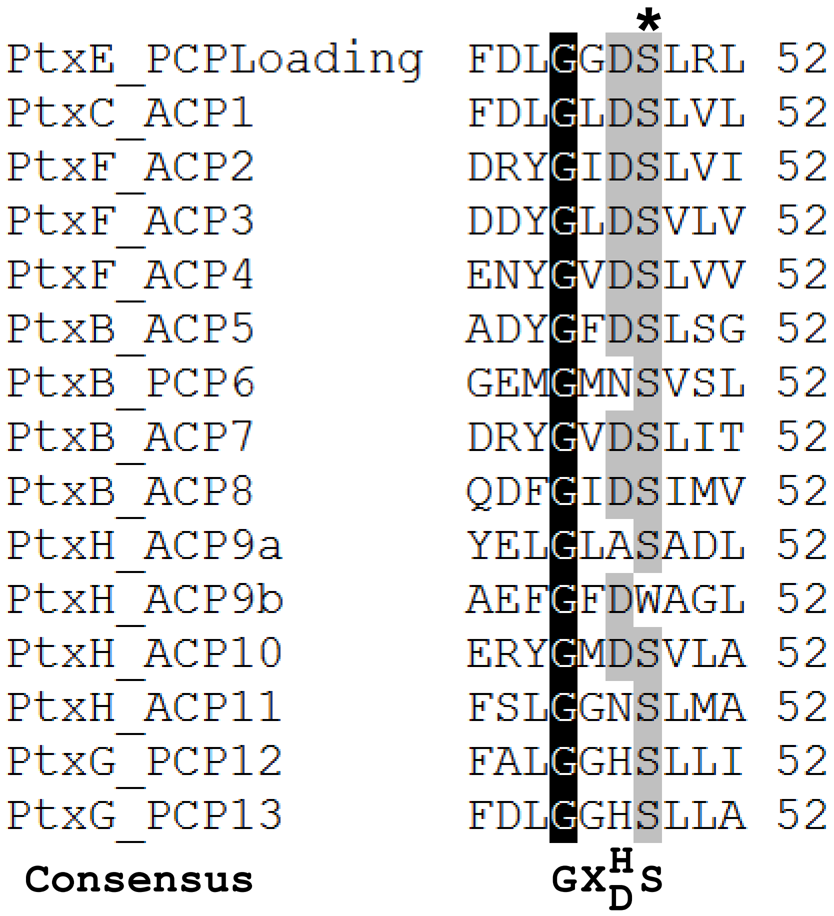

S4 Fig. Sequence alignment of the conserved motif in the ACP/PCP domain core regions from *ptx* PKSs and NRPSs. The Ser residue functioning as the phosphopantetheine-binding site is marked with an asterisk. The numbers indicate amino acid positions within each domain.
