# Supplementary material for: Characterization of the biosynthetic gene cluster for cryptic phthoxazolin A in Streptomyces avermitilis
Source: PLoS One. 2018 Jan 11;13(1):e0190973. doi: 10.1371/journal.pone.0190973 (PMC5764310; doi:10.1371/journal.pone.0190973)
Supplement: S5 Fig — The conserved catalytic residues are marked with asterisks. The core region for the NADP(H)-binding motif is underlined. The numbers indicate amino acid positions within each domain. The “LDD” motif for B-type KR domains is shown in a box. (PDF) [file pone.0190973.s005.pdf]

```
                                                 *
PtxF_KR2   VTGGAGGLGRAVADRLLS-RADARVVLLGR 33 GVVHAAG--VLHDGFALTKSEADFAAVLAPKVAGLR 107
PtxF_KR3   VTGGSGAIGRVLAEYLAR-TYRARLVLIGR 33 GVFHLAG--VADDGRATDGDRERFAAVLAAKTRGLV 116
PtxF_KR4   IIGGLGTVGRDTCRHLAQ-TYRAKLVVVGR 33 GVVNAAM--VLVDQVVRELPEAQLRSALDCKTESTW 116
PtxB_KR5   LIGGTGGIARELSLDLAR-RHRARLVWFSR 33 GVVHAAM--VFDNHPLAELDETAFTAATRVKIQGAT 116
PtxB_KR7   ITGGTGALGLLVAEHIAA-VRRAAGLSGGG 33 GILHAAG--VLRDGLLLRKDRADAEAVLAAKVHGTV 117
PtxB_KR8   ITGGTRGIGLLTARHFVAEHGVRKLVLTGR 35 GVVHSAGFTDFENPAFVRKPQSGMDRVIGPKVFGVD 135
PtxH_KR10  LTGGAGGLGLIVAREIAASVRRATVVLTGR 34 GILHAAG--VIEDNFVLRKSPEELERVLAPKVAGLV 117

consensus     GXGXXGXXXA

                             *         *   *
PtxF_KR2   ALDAATADDPLDFFVAFSSIAAHIGS--AGQTDYAYANAF 145
PtxF_KR3   HLDQLTRNDPLDLFVVFSSVSSLVGD--FGAASYATANRF 154
PtxF_KR4   SVLHAVRDESLDFVLFFSSGVAFEGN--HGQAGYAAGCTF 154
PtxB_KR5   ALAAAVAEERLDFLAFFSSAGSFGSF--AGNGAYICASAT 154
PtxB_KR7   LLDEATRDEPLDYFVAFSSAAAAFGN--AGQTDYAFANAF 155
PtxB_KR8   TLVEVFRDEPLRLFVLYSSVAATVPALAVGQSDYALANAY 175
PtxH_KR10  HLDEASRELPLEVFLCFSSLSGSFGN--PGQADYAAANAF 155
```

S5 Fig. Sequence alignment of the conserved motifs in the KR domain core regions from *ptx* PKSs. The conserved catalytic residues are marked with asterisks. The core region for the NADP(H)-binding motif is underlined. The numbers indicate amino acid positions within each domain. The “LDD” motif for B-type KR domains is shown in a box.
