# Supplementary material for: Characterization of the biosynthetic gene cluster for cryptic phthoxazolin A in Streptomyces avermitilis
Source: PLoS One. 2018 Jan 11;13(1):e0190973. doi: 10.1371/journal.pone.0190973 (PMC5764310; doi:10.1371/journal.pone.0190973)
Supplement: S1 Table — (PDF) [file pone.0190973.s007.pdf]

Table S1.

Oligonucleotides used in this study

Primer	Sequence (5' – 3')*
**For construction of large-deletion mutants**	
sav68-up-Fw	ATCATGAAGCTTATCCTCGTCCGGCTGCCCTGGTGGTGCGAG
sav68-up-Re	CCACTAGTGATATCGACGGTCTCCACCAGTGATGCGGACACACG
sav71-dw Fw	GCACTAGTTGGGCACTGTGGAGCGACGTGAGCCGGTG
sav71-dw-Re	ATCAGTAAGCTTTACACCAGACGCCGGTTCACGTTCCCCGCC
sav432-up-Fw	GAATTCAAGCTTCCCCCTGTCTCCGCACCGGCATCAGGAGGA
sav434-up-Re	GGACTAGTGGTGGTGACCAGGTACCTGTACAGGAAGCGCTCGCC
sav434-dw-Fw	GAATTCAAGCTTCCCCCTGTCTCCGCACCGGCATCAGGAGGA
sav434-dw-Re	TAATTCAAGCTTCTGAGGTCCGCAGGCGGGGCGGAGGTTCCG
sav845-up-Fw	ATGCAAAGCTTACGACGGAAGGAGCTGACCGCATG
sav845-up-Re	GGACTAGTGTAATAGGGGTCGTCCACCAGGTCTCGGC
sav845-dw-Fw	GCACTAGTCGAGCTGGAAGGAGGACACCGGCGGCATGGAC
sav845-dw-Re	CTGTCCAAGCTTACTGTTGCTCCCGAACGGCAGGTCCC
sav1007-up-Fw	GCATTCAAGCTTATGCTTTCGCACTCCATCGAGCAC
sav1007-up-Re	GGACTAGTGGTGATTGTCATCGACAGTTTCACCGTG
sav1007-dw-Fw	CGACTAGTTCGAGCCGACCTGGCTGACCACCCAGC
sav1007-dw-Re	GTGCAGAAGCTTGGCGCGGCTGGTGTAGATCCAGTTG
sav1286-up-Fw	CACTTCAAGCTTCCTTCCGGCATTTCGATGTGCCGCCC
sav1286-up-Re	GGACTAGTGATCATGGTCTTGTCGCCGGGCGCGGCTC
sav1286-dw-Fw	CGACTAGTCGACGCGCTCAGTGACTCGTTCGCCGC
sav1286-dw-Re	GAATTCAAGCTTACCTTCGGGCCCCCGGACACCTGG
wo-mutloxP-SpeI-Fw	GCACTAGTGCTCATTTAAATCCGTTGGATACACCAAG
wo-mutloxP-SpeI-Re	GGACTAGTTTAGACATTATTTGCCGGACTACCTTGGTGATCTCGCTTTCACGTAG
mutloxP-SpeI-Fw	GGACTAGTGAGCGACTCGAGTACCGTTCGTATAGCATACATTACGAAGTTATACGCG
mutloxP-SpeI-Re	CTCGAGACTAGTCTGGTACCGAGCGAACGCGTT
sav71-test-Fw	GGTCTCGGTCCTGACGGGGATGTCCACCC
sav71-test-Re	GACGTGTCGCTGAGCCGGTGGAAGGTGAC
sav434-test-Fw	TTCGCCCGCGCTCACTCCCCGTACTACC
sav434-test-Re	GGCTCTCGTTGCAGCCGTAGGTGTTGACCAC
sav1286-test-Fw	GACGAGGAGCTGCTGCGTCTGTGGG
sav1286 test-Re	GCGAGACCAGCACGATGCCGACGAG
sav845 test-Fw	GCTGCGACAGTGAAGGACGATCAGTGA

sav845 test- Rw	GTCGGCGGAGAAGAAGAACAAGCTGAC
**For construction of *ptxA* mutant and *ptxA*-complemented strain**	
ptxA-up-Fw	CGATCCAAGCTTAACGACTGCGTGTGCAGGTCGCCGCG
ptxA-up-Re	GCACTAGTCTGGAGGGCGCAGCCGCACACCTCATGGAG
ptxA-dw-Re	GGACTAGTGAACATGAACACGACGGGGAGGGAATCGGTG
ptxA-dw-Fw	CAATTCAAGCTTGCTCACCGACCGGCAGGGCCTCGACC
ptzA test-Fw	CAGTGTGTACGTCCAGACGGTCAGCG
ptzA test-Re	GCTCGAACTCAAGGCCGTGGAGTCG
hph-Fw	CGACTAGTCAGTGAGTTCGAGCGACTCGAGTACCGTTC
hph-Re	CTCGAGACTAGTCTGGTACCGAGCGAACGCGTT
ptzA-Fw	GCATCTCCTCGCGGGACTGCCTGCG
ptzA-Re	GGCAGGTTGCCGCGTGCGAAGTTG
ptxA-comp-Fw	GTGCCGGTTGGTAGTGGGAGGTGAATGAGGGCGCCAAGGGCCGCGCAGGC
ptxA-comp-Re	CTTTAGATTCTAGAGCCCGGGCAGGTGCGGTCAGCGGAAGGTACTCAAGCGC
apr-Fw	CCCCGGCGGTGTGCTG
apr-Re	GACGTCGCGGTGAGTTCAGGC

*Restriction sites are underlined.
